# Hyperfibrinolysis in Cats: A Retrospective Case–Control Study of 154 Cats

**DOI:** 10.1111/jvim.70201

**Published:** 2025-09-17

**Authors:** Anabel Priego‐Corredor, María Rocío del Saiz‐Álvarez, Anna Vila‐Soriano, Jorge Castro‐López, Vicente Herrería‐Bustillo

**Affiliations:** ^1^ Veterinary Teaching Hospital of the Catholic University of Valencia (VTHCUV) Valencia Spain; ^2^ AniCura Valencia Sur Veterinary Hospital, Internal Medicine Silla Spain

**Keywords:** cavitary effusion, coagulation, fibrinolysis, heart disease, liver disease, thromboelastography

## Abstract

**Background:**

Hyperfibrinolysis (HFL) has not been well described in cats.

**Hypothesis/Objectives:**

Identify risk factors associated with HFL in cats and evaluate its association with survival. Our hypotheses were that cats share similar conditions as dogs and humans with HFL and that it is associated with a worse prognosis.

**Animals:**

A total of 154 client‐owned cats had thromboelastography (TEG) performed because of a variety of clinical conditions.

**Methods:**

Retrospective case–control study. Cases were defined as cats with HFL (clot lysis at 30 min [LY30] ≥ 5% or clot lysis at 60 min [LY60] ≥ 10% or both) and controls were cats that had TEG performed but without HFL. Signalment, conditions that may alter fibrinolysis, TEG variables, indications to perform TEG, and outcome were recorded.

**Results:**

Fifty‐two cats (33.8%) with HFL and 102 controls (66.2%) were included. Demographic variables were similar between groups. Both liver disease (odds ratio [OR], 5.0; 95% confidence interval [CI], 2.4–10.6; *p* < 0.001) and the presence of cavitary effusion (OR, 5.9; 95% CI, 2.8–12.4; *p* < 0.001) were important risk factors for HFL. Cats with heart disease were less likely to have HFL (OR, 0.2; 95% CI, 0.1–0.5; *p* < 0.001). The presence of HFL was not associated with worse outcome (*p* = 0.84). Cats with HFL more often were hypocoagulable, whereas cats without HFL more often were hypercoagulable (*p* < 0.001).

**Conclusions and Clinical Importance:**

Liver disease and cavitary effusion are risk factors for HFL in cats. The presence of HFL did not affect survival.

**Abbreviations**
ALP: alkaline phosphate
ALT: alanine aminotransferase
APTEM: antifibrinolytic thromboelastometry
aPTT: activated partial thromboplastin time
ATC: acute traumatic coagulopathy
CI: confidence interval
DIC: disseminated intravascular coagulation
EXTEM: extrinsically activated thromboelastometry
FeLV: feline leukemia virus
FIBTEM: fibrinogen thromboelastometry
FIP: feline infectious peritonitis
FIV: feline immunodeficiency virus
FXIII: factor XIII
HFL: hyperfibrinolysis
IBD: inflammatory bowel disease
IMHA: immune‐mediated hemolytic anemia
LY30: clot lysis at 30 min
LY60: clot lysis at 60 min
MA: maximal amplitude
OR: odds ratio
PAI‐1: plasminogen activator inhibitor‐1
PROVETS: partnership on rotational viscoelastic test standardization
PT: prothrombin time
ROTEM: rotational thromboelastometry
TAFI: thrombin activatable fibrinolysis inhibitor
TEG: thromboelastography
tPA: tissue plasminogen activation
uPA: urokinase plasminogen activator

## 1 Introduction

Hemostasis is a complex system divided into three stages: primary hemostasis, secondary hemostasis, and fibrinolysis. The action of platelets and clotting factors forms a fibrin clot [1]. Factor XIII (FXIII) cross‐links the insoluble fibrin, stabilizing the clot [2]. Simultaneously, the process of fibrinolysis is initiated and eventually dissolves the thrombus [3]. Plasmin, generated from plasminogen by tissue plasminogen activator (tPA) and urokinase plasminogen activator (uPA), cleaves fibrin, forming soluble fibrin degradation products, including D‐dimers [4, 5, 6]. Hyperfibrinolysis (HFL) can cause premature clot lysis and bleeding, resulting from fibrinolysis protein abnormalities or excessive system activation [7, 8].

In humans, congenital HFL arises from deficiencies in alpha‐2‐antiplasmin and plasminogen activator inhibitor‐1 (PAI‐1), Quebec platelet disorder, hemophilia, FXIII deficiency, and fibrinogen disorders [9, 10, 11, 12, 13]. Hemophilia A also occurs in cats, but its impact on fibrinolysis requires further investigation [14, 15, 16, 17]. Fibrinogen disorders have been reported in veterinary medicine, but no direct relationship with HFL has been established [18, 19, 20, 21, 22]. Greyhounds with delayed postoperative bleeding show significantly decreased alpha‐2‐antiplasmin concentrations, suggesting enhanced fibrinolysis. Another study in healthy greyhounds using a point‐of‐care viscoelastic coagulometer showed increased fibrinolysis compared with non‐greyhound dogs [23, 24, 25]. A single case of FXIII deficiency with suspected HFL has been documented in a dog [26]. Rotational thromboelastometry (ROTEM) showed decreased maximum clot amplitude across assays, and a clot solubility test identified shortened lysis time compared with a control dog.

Acquired HFL in people is a consequence of disseminated intravascular coagulation (DIC), as well as being associated with end‐stage liver cirrhosis, liver transplantation, trauma, anaphylactic shock, neoplasia, and obstetric complications [27, 28, 29, 30, 31, 32]. In veterinary medicine, HFL has been reported in association with cavitary effusion, liver disease, DIC, and acute traumatic coagulopathy (ATC) [7, 33, 34, 35, 36, 37]. HFL also has been reported in 67% of dogs with *Angiostrongylus vasorum* infection and has been stimulated by the presence of *Dirofilaria immitis* antigens in dogs and cats [38, 39, 40]. HFL has been noted in cats with snake envenomation [7, 41].

Thromboelastography (TEG) assesses the viscoelastic properties of whole blood during clot formation and breakdown, but interpretation can be difficult because there is no established gold standard for evaluating fibrinolysis [9, 42, 43]. Clot strength reduction after maximal strength at 30 and 60 min is denoted as LY30 and LY60. Although there is no consensus on HFL diagnosis based on TEG, many references in the human and veterinary medical literature define HFL as LY30 ≥ 5% and LY60 ≥ 10% [37, 44, 45].

Our retrospective case–control study aimed to identify risk factors associated with HFL in cats by using TEG. A secondary goal was to evaluate whether HFL was associated with the duration of hospitalization and survival. We hypothesized that cats share similar HFL‐associated conditions with dogs and humans, and that the presence of HFL is associated with a worse prognosis and prolonged hospitalization.

## 2 Materials and Methods

The TEG database of our institution was retrospectively reviewed from October 2018 to March 2025. Our institution serves as a reference laboratory for nearby hospitals, and thus cats from multiple institutions were included in the study. All cats that underwent TEG analysis during the study period were evaluated for inclusion. Cases were defined as cats meeting predefined criteria for HFL (LY30 ≥ 5% or LY60 ≥ 10% or both). Additionally, all TEG traces were visually inspected to exclude technical artifacts, and those with ambiguous features were excluded. Controls were defined as cats that underwent TEG during the same period but did not fulfill the criteria for HFL.

The information recorded from each case included: signalment (age, sex, reproductive status, breed and weight), suspected conditions that may alter fibrinolysis (DIC, cavitary effusion, liver disease, neoplasia, ATC and cardiac disease), TEG variables (*R*, *K*, Angle, MA, *G*, LY30, LY60), and outcome (duration of hospitalization, survival to discharge, euthanasia, or natural death). Presumed indications to perform TEG and comorbidities also were recorded and classified using the international classification of diseases proposed by the World Health Organization. Cats were excluded if medical records were incomplete.

For our study, liver disease was defined as the presence of compatible clinical signs, abnormal hepatic ultrasonographic findings, a serum bilirubin concentration ≥ 3 mg/dL, and an increase in serum liver transaminase activities (alanine aminotransferase [ALT] or alkaline phosphatase [ALP]) or both > 2 times the upper reference limit. Disseminated intravascular coagulation was defined as the presence of thrombocytopenia and prolongation of both prothrombin time (PT) and activated partial thromboplastin time (aPTT) 1.5 times the upper reference limit and the existence of a predisposing factor such as sepsis, neoplasia, trauma, severe inflammation, or heat stroke. Acute traumatic coagulopathy was defined as a prolongation of clotting times (PT and aPTT) 1.5 times the upper reference limit or decreased clot strength (decreased MA or *G*) using viscoelastic methods in cats with severe trauma and hemorrhagic shock.

### 2.1 TEG Analysis

Blood samples were collected from three referral veterinary hospitals. All hospitals were within a 15‐km radius, and samples were transported at room temperature in citrate tubes (AQUISEL CI_3_Na 0.5 mL) to the central laboratory within 60 min of collection. All TEG analyses were performed at the same institution by a single operator, using the same machine (TEG 5000) and reagents (Haemonetics Kaolin), following an institutionally standardized protocol for sample handling and processing based on international guidelines [46, 47]. Samples were processed between 30 and 60 min after collection. Kaolin was used as an activator. The following TEG parameters were recorded: *R*, *K*, Angle, MA, *G*, LY30, and LY60. Reaction time (R) represents the time from initiation until the first fibrin polymers are produced, with a cut‐off value when the amplitude reaches 2 mm [42]. *K* time (K) measures the time until an amplitude of 20 mm is reached, indicating the speed of clot formation [42]. The slope of the line between *R* and *K* (α angle) represents the kinetics of fibrin production and cross‐linking [42]. The maximal amplitude (MA) denotes the maximal clot strength [42]. The *G* value represents the overall clot strength or global hemostatic potential and is calculated as follows: *G* = (5000 × MA)/(100 − MA) [42]. LY30 and LY60 represent the percentage of clot lysis detected at 30 and 60 min after reaching MA, respectively. Although the Partnership on Rotational ViscoElastic Test Standardization (PROVETS) group recommends creating site‐specific reference values for TEG analyses, in our study, we used reference ranges derived from published studies that employed the same TEG machine and reagents in cats [48, 49]. All cases in this study were analyzed using the same reference interval.

Additionally, all cats included in the study were categorized as hypercoagulable, normocoagulable, or hypocoagulable depending on their overall clot strength based on previously published information [50]. A hypocoagulable state was defined when *G* was < 4.24 kdyne/cm^2^, normocoagulable when *G* was 4.24–10.32 kdyne/cm^2^, or hypercoagulable when *G* was > 10.32 kdyne/cm^2^.

### 2.2 Statistical Analysis

The distribution of continuous data was assessed using the Shapiro–Wilk test. Normally distributed data are presented as mean ± SD, whereas non‐normally distributed data are presented as median and range. Categorical data are described as numbers and percentages. Comparisons between cases and controls were performed using the Mann–Whitney *U* test for continuous variables and the Chi‐squared test for categorical variables. Odds ratios (OR) with 95% confidence intervals (CIs) were calculated to evaluate associations between HFL and potential risk factors. A *p* value < 0.05 was considered statistically significant for all analyses. Commercially available statistical software was used for the statistical analysis (IBM SPSS Statistics Base V27).

## 3 Results

A total of 154 cats were included in the analysis, with case distribution across contributing hospitals as follows: Veterinary Teaching Hospital of the Catholic University of Valencia (*n* = 149, 96.7%), AniCura Valencia Sur Veterinary Hospital (*n* = 3, 2%), and Archiduque Carlos Veterinary Hospital (*n* = 2, 1.3%). Fifty‐two cats (33.8%) fulfilled the diagnostic criteria for HFL and were classified as cases. The remaining 102 cats (66.2%) were classified as controls, which corresponds to a prevalence of HFL of 33.8% among the studied population. A post hoc sample size estimation indicated that detecting a 10% absolute difference in exposure prevalence (at alpha 0.05 and 80% power) would have required approximately 388–392 cats. Given the study's sample size of 154 cats, it is likely adequately powered to detect moderate‐to‐large associations, but may be underpowered for small effect sizes.

The demographic variables of the overall sample, separated into cases and controls, can be found in Table 1. No significant differences were found between both groups. The most common breed in both groups was domestic shorthair, with 77 cats (75.5%) in the control group and 41 cats (78.8%) in the case group. Among cats with HFL, additional breeds included: Siamese (*n* = 4, 7.7%), exotic shorthair (*n* = 2, 3.8%), British shorthair (*n* = 1, 1.9%), Persian (*n* = 1, 1.9%), Sphynx (*n* = 1, 1.9%), Birman (*n* = 1, 1.9%), and Norwegian Forest (*n* = 1, 1.9%). Among cats without HFL, other breeds included: Persian (*n* = 8, 7.8%), British shorthair (*n* = 5, 4.9%), Siamese (*n* = 5, 4.9%), Sphynx (*n* = 2, 2%), Ragdoll (*n* = 2, 2%), Maine Coon (*n* = 1, 1%), and Norwegian Forest (*n* = 1, 1.9%). No significant association was found between breed and the presence of HFL (*p* = 0.23).

**TABLE 1:** Comparison of demographic variables in the study population of 154 cats between cases with hyperfibrinolysis (*n* = 52) and controls without hyperfibrinolysis (*n* = 102) as assessed via thromboelastography.

Demographic variables		Total (*n* = 154)	Cases (*n* = 52)	Controls (*n* = 102)	*p*
Age (years) Median (range)		10 (0.3–18)	9 (0.3–17)	10 (0.5–18)	0.12
Sex (*n*)	M	81 (56.2%)	26 (50%)	55 (53.9%)	0.64
	F	73 (47.4%)	26 (50%)	47 (46.1%)	0.64
Reproductive status (*n*)	E	15 (9.7)	6 (11.5%)	9 (8.8%)	0.59
	N	139 (90.3)	46 (88.5%)	93 (91.2%)	0.59
Weight (kg) Median (range)		4 (1.8–9)	4.3 (2.2–9)	4 (1.8–6.9)	0.6

Abbreviations: E, entire; F, female; M = male; N = neutered.

Table 2 describes conditions that may have prompted clinicians to perform TEG. The most common presumed indications for TEG testing were liver disease and cardiovascular disease, occurring in 45 cats for both conditions (29.2% of the total). Reported liver diseases included cholangiohepatitis and hepatic lipidosis. Reported neoplasms were lymphoma (nasal, mediastinal, and alimentary), carcinoma (cholangiocellular and mammary), and acute leukemia. Cardiovascular diseases included hypertrophic cardiomyopathy, restrictive cardiomyopathy, and unclassified cardiomyopathy. Endocrine diseases included hyperthyroidism, diabetes mellitus, and hyperaldosteronism. Reported neurological conditions were peripheral vestibular disease, seizures, and suspected ischemic cerebrovascular disease. Infectious diseases included feline infectious peritonitis (FIP), feline leukemia virus (FeLV), feline immunodeficiency virus (FIV), *Leishmania* spp., and *Mycoplasma* spp. The only hemolymphatic disease reported was immune‐mediated hemolytic anemia (IMHA), and the only gastrointestinal disease reported was inflammatory bowel disease (IBD).

**TABLE 2:** Comparison of conditions that may have prompted clinicians to perform TEG in the study population of 154 cats.

Presumed indications to perform TEG	Cases (*n* = 52), *N* (%)	Controls (*n* = 102), *N* (%)
Liver disease	27 (51.9%)	18 (17.6%)
Neoplasia	14 (26.9%)	17 (16.7%)
Cardiovascular disease	6 (11.5%)	39 (38.2%)
Genitourinary disease	5 (9.6%)	18 (17.6%)
Endocrine disease	1 (1.9%)	10 (9.8%)
Neurological disease	0	8 (7.8%)
Infectious disease	2 (3.8%)	3 (2.9%)
Hemolymphatic disease	0	7 (6.9%)
Gastrointestinal disease	0	4 (3.9%)

*Note:* The cases represent cats with hyperfibrinolysis (*n* = 52) and the control cats without hyperfibrinolysis (*n* = 102).

To evaluate possible risk factors for HFL, suspected conditions that may alter fibrinolysis were compared between cases and controls (Table 3). Cats with HFL were most commonly presented with cavitary effusions (55.8% vs. 17.6%; OR, 5.9; 95% CI, 2.4–10.6; *p* < 0.001) or liver disease (50% vs. 17.6%; OR, 5.0; 95% CI, 2.8–12.4; *p* < 0.001) compared with controls, suggesting that these are important risk factors for HFL in cats. In contrast, cardiac disease was significantly less common in cats with HFL (11.5% vs. 38.2%; OR, 0.2; 95% CI, 0.1–0.5; *p* < 0.001). No significant association was found between HFL and neoplasia (25% vs. 16.7%; OR, 1.8; 95% CI, 0.8–4.1; *p* = 0.13). Only one cat had DIC; this isolated occurrence precludes definitive conclusions about DIC as a risk factor. No cases of ATC were identified in either group.

**TABLE 3:** Comparison of conditions that may alter fibrinolysis between cats with hyperfibrinolysis (*n* = 52) and without hyperfibrinolysis (*n* = 102) as assessed via thromboelastography.

Conditions that may alter fibrinolysis	Cases (*N* = 52)	Controls (*N* = 102)	*p*	Odds ratio	95% CI
Cavitary effusion	29 (55.8%)	18 (17.6%)	< 0.001	5.9	2.8–12.4
Liver disease	26 (50%)	18 (17.6%)	< 0.001	5.0	2.4–10.6
Neoplasia	13 (25%)	17 (16.7%)	0.13	1.8	0.8–4.1
Cardiac disease	6 (11.5%)	39 (38.2%)	< 0.001	0.2	0.1–0.5

Cats with HFL more often were hypocoagulable compared with controls (19.2% vs. 5.9%; *p* < 0.001) whereas cats without HFL more often were hypercoagulable compared with cases (78.4% vs. 36.5%; *p* < 0.001). Among cats with HFL, 23 (44.2%) were normocoagulable, 19 (36.5%) were hypercoagulable, and 10 (19.2%) were hypocoagulable. In contrast, among cats without HFL, 16 (15.7%) were normocoagulable, 80 (78.4%) were hypercoagulable, and 6 (5.9%) were hypocoagulable. Thromboelastographic variables also were compared between cases and controls (Table 4). Cats with HFL had significantly lower MA (62.8 vs. 72.6 mm; *p* < 0.001) and *G* (8431.3 vs. 13236.2 dyne/cm^2^; *p* < 0.001) compared with controls, consistent with their more hypocoagulable profile, whereas controls were predominantly hypercoagulable.

**TABLE 4:** Comparison of thromboelastographic variables in the study population of 154 cats.

TEG variables	Cases (*n* = 52)	Controls (*n* = 102)	*p*	95% CI
*R* (min)	3.2 (0.2–11.2)	3.8 (1.2–85.1)	0.04	0–1.1
*K* (min)	1.2 (0.1–13.7)	1.1 (0.1–20)	0.5	−0.2‐0.1
Angle (deg)	73.6 (14.9–82.9)	73.7 (7.6–87.5)	0.9	−2.4‐2.6
MA (mm)	62.8 (4.2–87.2)	72.6 (4.3–86.8)	< 0.001	5.2–13.9
*G* (dyne/cm^2^)	8431.3 (5.8–34048.2)	13236.2 (227–33004.8)	< 0.001	2662.5–6637.9
LY30 (%)	11 (0.3–92.1)	0 (0–4.6)	< 0.001	8.2–13.6
LY60 (%)	21.9 (10.4–91.4)	1.7 (0–9.1)	< 0.001	16.6–25.9

*Note:* The cases represent cats with hyperfibrinolysis (*n* = 52) and the controls represent cats without hyperfibrinolysis (*n* = 102). All variables are reported as median (range). Reference intervals used for comparison in this table are based on previously published data and were not established using a healthy reference population within this study.

Abbreviations: CI, confidence interval; *G*, global clot strength; *K*, clot formation time; LY30, percentage of clot lysis 30 min post MA; LY60, percentage of clot lysis 60 min post MA; MA, maximum amplitude; *N*, number of cats; *R*, reaction time.

Regarding the duration of hospitalization, no significant differences were found between cases (4 days [0–16]) and controls (3 days [0–16]; *p* = 0.05). Survival to hospital discharge also was similar between cases (39 cats [75%]) and controls (75 cats [73.5%]; *p* = 0.84).

## 4 Discussion

The results of our retrospective case–control study suggest that cavitary effusion and liver disease are important risk factors for HFL in cats, whereas heart disease may be associated with a lower likelihood of developing HFL. Hyperfibrinolytic cats more often were hypocoagulable compared with cats without HFL, whereas cats without HFL more often were hypercoagulable. HFL was not associated with a worse outcome.

Previously published studies have reported that 40% and 50% of dogs with HFL presented with pleural and peritoneal effusion, respectively, and those effusions were secondary to a variety of causes [35, 36]. In another study, it was reported that 50% of cats with enhanced fibrinolytic activity had hemorrhagic effusions [7]. These results were similar to those obtained in our study, supporting the existence of a pathophysiological relationship between cavitary effusion and HFL. This association may be explained by the increased fibrinolytic activity of mesothelial cells of the pericardium, pleural space, and peritoneum, which are important regulators of fibrin concentrations when an injury within a serosal cavity occurs [51]. Mesothelial cells secrete tPA and uPA, activating plasmin, which in turn cleaves fibrin in the pericardial, pleural, and peritoneal fluid [52, 53]. This normal fibrinolytic activity may become upregulated in the presence of effusions, contributing to the observed association with HFL. Additionally, mesothelial cells can enhance anticoagulation by increasing local expression of protein C [54]. Systemic HFL may occur when fluid rich in fibrinolysis mediators is reabsorbed into the lymphatic circulation and returned to the systemic circulation through the thoracic duct, secondary to cavitary effusion [55].

In our study, liver disease was a common indication for TEG testing. This observation may be attributed to the fact that individuals with liver disease often present with complex and evolving coagulation abnormalities [37]. TEG has proven useful in identifying the potential hemorrhagic and prothrombotic tendencies of these patients [37]. Previous studies in people and dogs with liver disease have identified alterations in the fibrinolytic system that can result in either a hypo‐ or hyperfibrinolytic state [30, 37, 56]. A study of dogs with acute liver disease identified 38% as hyperfibrinolytic, and LY30 showed positive correlations with serum bilirubin concentrations and white blood cell count, and was negatively correlated with serum cholesterol concentrations [37]. Another study identified that 23.8% of dogs with chronic hepatopathies had HFL, which was associated with high disease activity (significantly higher serum transaminase activities) [45]. In a study of cats with cholestatic liver disease, 28% were hyperfibrinolytic, and LY60 was positively correlated with PT [50]. HFL occurs in these cases because of decreased hepatic production of antifibrinolytic proteins such as alpha 2‐antiplasmin and decreased hepatic clearance of plasminogen activators and plasmin [30]. In addition, cavitary effusions and DIC, conditions that often are associated with severe liver disease as observed in our study, may result in systemic primary HFL. Furthermore, deficiency in factor XIII has been associated with HFL because this factor is responsible for stabilizing the fibrin clot, and therefore, its deficiency could lead to premature clot lysis. A previous study found that up to 70% of cats with liver disease had low FXIII concentrations [57].

In our study, neoplasia was not identified as a significant risk factor for HFL in cats. This finding could be true, or it could represent a type II statistical error because our study likely was underpowered to detect small effect sizes. A recent systematic review indicated that HFL as a paraneoplastic phenomenon in human patients with solid malignant neoplasms is a rare condition, described in a limited number of case studies [58]. In veterinary medicine, HFL associated with neoplasia remains poorly characterized in dogs and has not been documented in cats. One prospective case–control study identified clinically relevant HFL in dogs with sarcomas, but the underlying mechanisms remain unclear [59]. Multicentric lymphoma‐associated HFL also has been observed exclusively in dogs with stage V disease, suggesting its potential use as a marker of advanced disease severity [60]. Interpretation of these data is complicated by the frequent coexistence of DIC [61, 62]. Whereas studies of humans and dogs have established associations between specific malignancies and HFL, our study of cats identifies a distinct clinical paradigm. Additional studies incorporating larger sample sizes encompassing diverse tumor types and utilizing advanced staging techniques are required to definitively exclude neoplasia as a risk factor for HFL in cats, particularly for tumor types underrepresented in our study.

Our results identified that cats with heart disease were less likely to have HFL. However, because TEG alone cannot determine whether fibrinolysis is merely normal or truly decreased, diagnosing hypofibrinolysis would require measurement of specific fibrinolytic and antifibrinolytic mediators, which was not performed in our study. Nevertheless, evidence from cardiology in human patients shows that several cardiac conditions (particularly degenerative mitral valve disease, aortic stenosis, and atrial fibrillation) are accompanied by increased concentrations of antifibrinolytic factors (including PAI‐1 and thrombin activatable fibrinolysis inhibitor [TAFI]) which collectively decrease endogenous fibrinolytic activity and promote prothrombotic states [63, 64]. In cats, the relationship between cardiac disease and fibrinolysis remains poorly characterized. Current pathophysiological models for arterial thromboembolism (ATE) emphasize endothelial damage, platelet activation, coagulation factor activation, and blood stasis caused by left atrial enlargement and impaired contractility as key contributors [65]. A recent study showed that the risk of ATE in cats with cardiogenic pleural effusion was significantly lower (6.6%) than in cats with cardiogenic pulmonary edema (33.3%) or in those with cardiac disease without evidence of congestive heart failure (15.6%) [66]. This finding suggests that cardiogenic pleural effusion may be protective against ATE. As previously noted, cavitary effusions contain proteins and enzymes involved in coagulation and fibrinolysis, resulting in fluid with inherently increased fibrinogenolytic and fibrinolytic activity [67]. Therefore, the decreased risk of ATE in cats with cardiogenic pleural effusion, compared with cats with cardiogenic pulmonary edema or cardiac disease without signs of heart failure, may reflect a decreased capacity for thrombus formation because of increased systemic fibrinolytic activity triggered by reabsorption of pleural fluid into the circulation [66]. An alternative hypothesis is that these cats have less systemic inflammation because inflammatory and hemostatic pathways are closely linked and contribute to thrombus formation in the context of inflammation [68]. Our findings, which show that cats with cavitary effusions were more likely to exhibit HFL, whereas those with cardiac disease were less likely to show HFL, support the hypothesis that decreased likelihood of HFL in cardiac patients may be an underrecognized contributor to thrombotic risk and that effusion‐associated enhancement of fibrinolytic activity may offer some protective effect.

Our study indicates that cats with HFL frequently exhibit a hypocoagulable profile, whereas those without HFL tend toward hypercoagulability. These findings are consistent with previous reports in both human and veterinary medicine, which suggest that excessive fibrinolytic activity can compromise clot stability and contribute to a hypocoagulable state [2]. One study investigating HFL in cats and dogs using ROTEM found that HFL was associated with prolonged clotting times in both extrinsically activated thromboelastometry (EXTEM) and antifibrinolytic thromboelastometry (APTEM) assays, as well as decreased maximum clot firmness (MCF) in fibrinogen thromboelastometry (FIBTEM), compared with reference intervals. In dogs, these findings were further accompanied by decreased EXTEM MCF and maximum clot elasticity, indicating that HFL impairs the formation of stable clots and leads to hypocoagulability in addition to earlier clot lysis [7]. Similarly, in cats with cholestatic liver disease, HFL has been associated with thromboelastographic evidence of hypocoagulability [50]. This pattern may reflect advanced disease stages and loss of synthetic function, DIC, or persistent vitamin K deficiency [50]. Furthermore, one study showed that dogs with chronic inflammatory enteropathy were hypercoagulable and hypofibrinolytic compared with healthy controls, as assessed by TEG [69]. These findings mirror observations in humans with inflammatory bowel disease (IBD), in whom hypofibrinolysis is reported frequently. Notably, the presence of anti‐tPA antibodies has been documented in some human IBD patients, and development of these antibodies has been proposed as a potential mechanism underlying the observed hypofibrinolytic and prothrombotic state in this population [70]. Taken together, our results suggest that fibrinolytic and coagulation pathways may not always counterbalance each another to minimize thrombotic or hemorrhagic risk. Instead, HFL may compromise clot stability and amplify hypocoagulability, increasing the risk of bleeding, whereas the coexistence of hypofibrinolysis and hypercoagulability may create a particularly thrombogenic environment. This lack of physiological cross‐regulation emphasizes the importance of comprehensive hemostatic assessment (including both coagulation and fibrinolytic parameters) to accurately evaluate the risk of thromboembolic or hemorrhagic events in feline patients.

Viscoelastic tests provide a comprehensive assessment of the coagulation system by detecting changes in blood viscosity during different coagulation phases [42]. The use of TEG has been considered a reproducible method for evaluating hemostasis in veterinary medicine [71]. HFL is evaluated by the parameters LY30 and LY60. Although attempts have been made to define reference ranges for viscoelastic testing, values reflecting HFL still need to be standardized and well‐defined. Studies in human medicine have considered TEG values as indicative of HFL when LY30 ≥ 3% in trauma patients and LY30 > 7.5% in the remaining patients [9]. There is no consensus in veterinary medicine, and published reports have considered HFL with LY30 values between 3% and 7% and LY60 values between 10% and 15% [7, 37, 45, 50]. However, caution is necessary when defining HFL solely based on increases above these reference intervals. Such thresholds may represent normal variations rather than pathological fibrinolytic activity, similar to how coagulopathies are defined using specific thresholds for clotting times. A study using ROTEM in healthy cats established average values for LY30 between 0% and 9% and expected values for LY60 between 1% and 13% [72]. Although ROTEM is considered more sensitive to fibrinolysis than TEG, the two tests are not interchangeable, as noted in the PROVETS guidelines [49]. Based on all of these studies and considering the limitations, we used a threshold of LY30 ≥ 5% or LY60 ≥ 10% or both to define HFL in our study. It is also important to acknowledge that unmodified TEG has recognized limitations in detecting mild‐to‐moderate increases in fibrinolysis in both humans and dogs [73]. These limitations may have resulted in an underestimation of hyperfibrinolytic cases in our study. To improve sensitivity, modified TEG protocols incorporating exogenous fibrinolytic triggers (i.e., tPA‐TEG) have been developed [59, 74, 75, 76, 77]. However, tPA‐TEG has not yet been validated for use in cats, and was not available for use in our study. These findings emphasize the need for standardized diagnostic criteria and additional research into cat‐specific fibrinolysis assays.

In our investigation, survival to hospital discharge was 74% and there was no significant association between HFL and outcome in cats. Previous studies of people with trauma have suggested that HFL is a strong predictor of early in‐hospital mortality and massive transfusion. Another study suggested that increased fibrinolysis at admission predicts poor outcome in cardiac arrest with presumed cardiac etiology in humans [78]. Our results were similar to those from another study of cats with HFL that reported 63% survival to hospital discharge and found no association between HFL and outcomes or bleeding events [7]. Notably, no cats in our study underwent TEG analysis specifically for trauma evaluation, and consequently, ATC was not included. This limitation prevented the evaluation of ATC as a risk factor for HFL.

Although our case–control study provided relevant data on HFL in cats, it had several limitations. First, the control group was not composed of standardized or healthy cats. Instead, it included patients undergoing TEG for various clinical indications but without evidence of HFL, which may introduce heterogeneity and add complexity to the interpretation of findings. Second, the retrospective design inherently limited data collection. As a result, important variables that could influence TEG results (e.g., concurrent treatment or prior blood transfusions) could not be fully considered. Third, there is a potential for inclusion bias. For instance, TEG was not performed in cats with certain conditions (e.g., trauma) that are suspected to influence fibrinolysis, potentially leading to an underrepresentation of important risk factors for HFL. Fourth, fibrinogen (a key factor in coagulation) was not measured in all cats, which may have limited our ability to further characterize cases of HFL. Fifth, the absence of standardized diagnostic criteria for HFL, along with the known tendency of cats to be hyperreactive, may have contributed to hyperfibrinolytic‐like TEG patterns. Indeed, some previous studies have reported HFL even in apparently healthy cats, raising concerns about potential overdiagnosis [72, 79, 80]. Sixth, we could not identify cats with hypofibrinolysis because we did not measure specific fibrinolysis mediators. Thromboelastography alone is not a valid method to diagnose hypofibrinolysis. Seventh, the use of post hoc sample size calculations introduces uncertainty regarding the study's statistical power. Because these calculations were based on observed data rather than predefined hypotheses, they offer only an approximate estimate and are inherently prone to bias. As a result, the reliability of conclusions (particularly those involving weaker associations) may be limited by insufficient power. Further research involving larger sample sizes, prospective study designs, standardized healthy controls, and the development of validated diagnostic criteria (potentially including tPA‐modified TEG protocols) is essential to improve the detection, understanding, and clinical relevance of HFL in cats.

## 5 Conclusions

We identified cavitary effusion and liver disease as important risk factors for the development of HFL in cats. In contrast, cats with heart disease were less likely to have HFL. Furthermore, cats with HFL more often were hypocoagulable, whereas cats without HFL tended to be hypercoagulable. In this population, survival to hospital discharge of 74% was reported, and the presence of HFL was not associated with a worse outcome.

## Disclosure

Authors declare no off‐label use of antimicrobials.

## Ethics Statement

Authors declare no institutional animal care and use committee or other approval was needed. Authors declare human ethics approval was not needed.

## Conflicts of Interest

The authors declare no conflicts of interest.
